# Optimizing Miniscrew Stability: A Finite Element Study of Titanium Screw Insertion Angles

**DOI:** 10.3390/biomimetics10100650

**Published:** 2025-10-01

**Authors:** Yasin Akbulut, Serhat Ozdemir

**Affiliations:** 1 Department of Orthodontics, Kocaeli Health and Technology University, 41275 Kocaeli, Turkey; 2 Department of Orthodontics, Private Clinics, 63320 Sanliurfa, Turkey; dt.serhat@hotmail.com

**Keywords:** orthodontic miniscrew, finite element analysis, insertion angle, cortical bone stress, primary stability, biomechanics

## Abstract

This study aimed to evaluate how different insertion angles of titanium orthodontic miniscrews (30°, 45°, and 90°) influence stress distribution and displacement in surrounding alveolar bone using three-dimensional finite element analysis (FEA), with a focus on biomechanical outcomes at the titanium–bone interface. The 90° insertion angle generated the highest stress in cortical bone (58.2 MPa) but the lowest displacement (0.023 mm), while the 30° angle produced lower stress (36.4 MPa) but greater displacement (0.052 mm). The 45° angle represented a compromise, combining moderate stress (42.7 MPa) and displacement (0.035 mm). This simulation-based study was conducted between January and April 2025 at the Department of Orthodontics, Kocaeli Health and Technology University. A standardized 3D mandibular bone model (2 mm cortical and 13 mm cancellous layers) was constructed, and Ti-6Al-4V miniscrews (1.6 mm × 8 mm) were virtually inserted at 30°, 45°, and 90°. A horizontal orthodontic load of 2 N was applied, and von Mises stress and displacement values were calculated in ANSYS Workbench. Stress patterns were visualized using color-coded maps. The 90° insertion angle generated the highest von Mises stress in cortical bone (50.6 MPa), with a total maximum stress of 58.2 MPa, followed by 45° (42.7 MPa) and 30° (36.4 MPa) insertions (*p* < 0.001). Stress was predominantly concentrated at the cortical entry point, especially in the 90° model. In terms of displacement, the 90° group exhibited the lowest mean displacement (0.023 ± 0.002 mm), followed by 45° (0.035 ± 0.003 mm) and 30° (0.052 ± 0.004 mm), with statistically significant differences among all groups (*p* < 0.001). The 45° angle showed a balanced biomechanical profile, combining moderate stress and displacement values, as confirmed by post hoc analysis. From a biomimetics perspective, understanding how insertion angle affects bone response provides insights for designing bio-inspired anchorage systems. By simulating natural stress dissipation, this study demonstrates that insertion angle strongly modulates miniscrew performance. Vertical placement (90°) ensures rigidity but concentrates cortical stress, whereas oblique placement, particularly at 45°, offers a balanced compromise with adequate stability and reduced stress. These results emphasize that beyond material properties, surgical parameters such as insertion angle are critical for clinical success.

## 1. Introduction

Orthodontic miniscrews, a type of temporary anchorage device (TAD), revolutionized fixed anchorage control in appliance therapy by offering a simple, minimally invasive, and highly efficient solution that does not rely on patient compliance [1,2,3,4]. Their introduction significantly enhanced the ability of clinicians to perform controlled tooth movements such as molar distalization, en-masse retraction, vertical control, and space closure with improved anchorage reliability [1,5,6,7]. Unlike conventional anchorage methods that depend on teeth, extraoral appliances, or patient cooperation, miniscrews provided absolute anchorage directly from the bone [8,9].

Despite their numerous advantages, the success of miniscrews was influenced by multiple biomechanical and anatomical variables, including bone density, cortical bone thickness, miniscrew design, insertion site, and insertion angle [6,7,10,11,12,13]. Among these, the angle of insertion emerged as a key factor in determining the initial stability and stress distribution around the miniscrew-bone interface [14,15,16,17]. Studies demonstrated that oblique insertion angles, typically ranging from 30° to 60°, increased the contact surface between the screw threads and the cortical bone, thereby improving mechanical interlocking and potentially enhancing primary stability [18,19,20]. Moreover, oblique placement was believed to reduce the risk of root damage by altering the trajectory of insertion in anatomically constrained sites [21,22].

Biomimetics, the discipline that translates strategies from biological systems into engineering and medical innovations, has profoundly influenced the development of dental and orthopedic implants [23,24]. The alveolar bone–implant interface itself represents a highly specialized biomechanical environment where stress distribution, remodeling capacity, and adaptive responses resemble broader biological optimization processes observed in skeletal tissues [25]. By analyzing how variations in insertion angle alter stress concentration patterns, our study draws parallels to natural load-bearing adaptations, thereby situating miniscrew research within the biomimetic paradigm.

In this context, finite element analysis (FEA) serves not only as a computational tool but also as a bridge between engineering and biology, enabling the extraction of bio-inspired principles from simulated bone–implant interactions [26,27]. Insights from this study extend beyond orthodontics: they provide a model for how biomimetic approaches can inform the design of skeletal anchorage systems that emulate the balance between rigidity and flexibility observed in natural bone structures [28,29]. This strengthens the relevance of our findings to the journal’s biomimetics focus and underscores their translational potential in both dental and broader biomedical applications.

However, the biomechanical implications of angled insertion remained controversial. Some authors suggested that steeper angles might lead to excessive stress concentrations at the cortical bone layer, especially under orthodontic loading conditions, which in turn could predispose to microdamage, bone resorption, or early implant loosening [5,30]. Furthermore, the variability in bone morphology and screw design complicated the generalization of clinical recommendations. Therefore, precise understanding of how stress is distributed in cortical and trabecular bone tissues at different insertion angles became crucial for optimizing the clinical use of miniscrews [31,32].

Finite element analysis (FEA) has proven to be a valuable computational tool for simulating the mechanical behavior of complex structures such as bone-implant systems under controlled loading scenarios [33,34,35,36]. By creating accurate three-dimensional (3D) models and applying realistic forces and boundary conditions, FEA allowed researchers to predict stress distributions, deformations, and failure points in a virtual environment, without subjecting patients to risk [22,37,38]. Although FEA studies have extensively evaluated the effects of miniscrew dimensions, thread pitch, and bone density on mechanical stability [39,40], there has been a lack of detailed analysis focusing on the role of insertion angle in altering stress patterns within both cortical and spongy bone under orthodontic force application. The influence of insertion angle on miniscrew stability has long been recognized. Early works such as Wilmes et al. and Laursen et al. demonstrated that oblique placement increases cortical contact and reduces the risk of root damage [19,20]. Likewise, Kuroda et al. emphasized root proximity as a major factor in miniscrew failure, highlighting the importance of insertion trajectory [41]. These foundational studies continue to inform contemporary finite element analyses.

Recent systematic reviews and meta-analyses have further underscored the role of insertion parameters, surface modifications, and patient-related variables in determining miniscrew success rates [2,3,4,6,7]. However, despite extensive research, the biomechanical impact of insertion angle has remained an area of debate, with FEA emerging as a powerful tool to simulate these mechanical interactions [10,13,15].

This study aimed to investigate the effect of three different insertion angles—30°, 45°, and 90°—on the stress distribution around orthodontic miniscrews using three-dimensional finite element modeling. By identifying the biomechanical advantages and potential risks associated with each angulation, this research sought to provide evidence-based guidance for clinicians regarding optimal insertion strategies to improve miniscrew performance and reduce the risk of failure.

## 2. Materials and Methods

### 2.1. Study Design and Patient Selection

This simulation-based study was conducted between January and April 2025 at the Department of Orthodontics, Kocaeli Health and Technology University. It was designed as a three-dimensional finite element analysis (FEA) to investigate the effect of different miniscrew insertion angles on stress distribution in the surrounding alveolar bone under orthodontic loading. As the study did not involve human or animal subjects, ethical approval was not required.

### 2.2. Finite Element Model Construction

#### 2.2.1. Bone and Miniscrew Modeling

**Figure 1:**
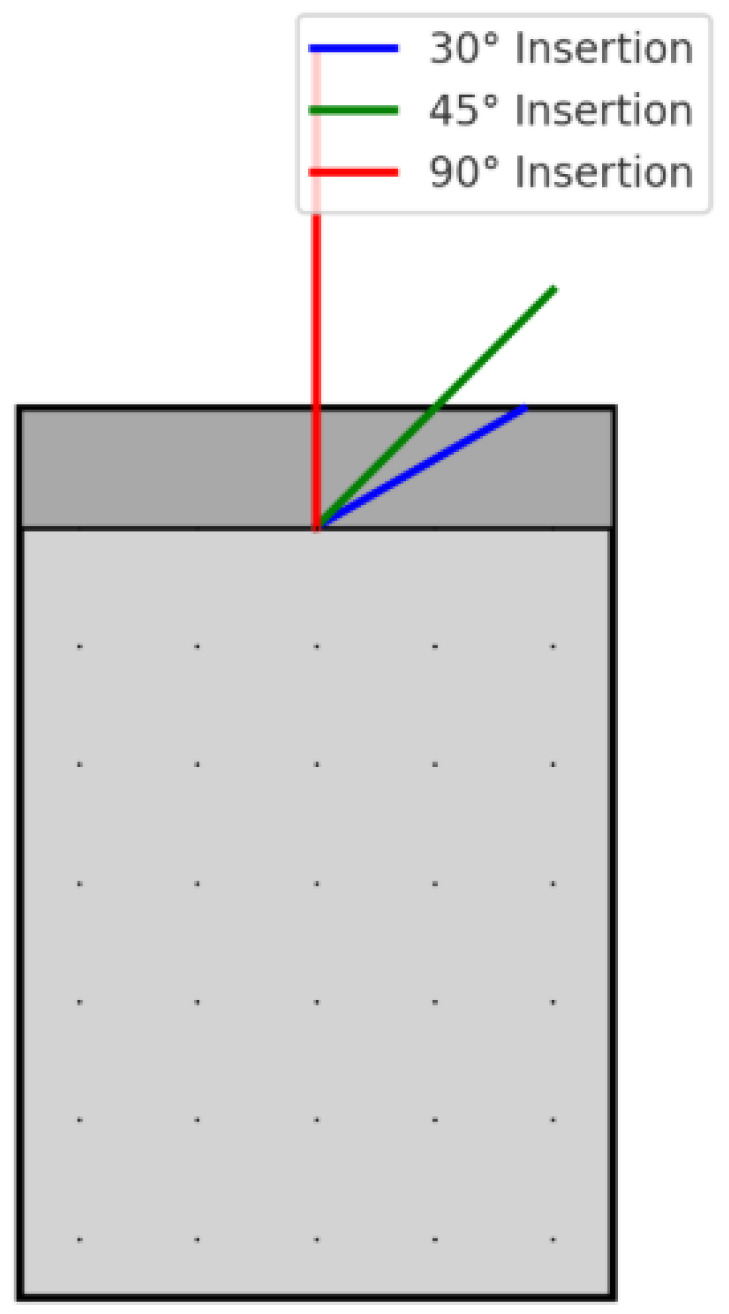
Virtual bone block with cortical (dark grey) and cancellous (light grey) layers. Miniscrew insertion angles: 30° (blue), 45° (green), 90° (red). Mesh representation shown as dots.

A virtual segment of mandibular alveolar bone was modeled in SolidWorks 2023 (Dassault Systèmes, Vélizy-Villacoublay, France) based on published anatomical and FEA references [41,42,43]. The model consisted of a rectangular block (10 × 10 × 15 mm = 1500 mm^3^) with an outer 2 mm cortical layer and an inner 13 mm cancellous core, reflecting typical mandibular bone proportions. The cortical and cancellous bone were modeled as isotropic, homogeneous, and linearly elastic structures to ensure computational feasibility (Figure 1).

A titanium orthodontic miniscrew was also modeled to simulate a commonly used clinical design. The screw had a total length of 8 mm and a diameter of 1.6 mm, with a thread pitch of 0.8 mm. Its geometry included a cylindrical body with a tapered apical tip, representing a self-drilling configuration frequently employed in clinical practice. The material assigned to the screw was Ti-6Al-4V titanium alloy, a biocompatible and widely used alloy in dental and orthopedic applications due to its high strength and corrosion resistance.

Three separate models were created to simulate different insertion angles of the miniscrew into the bone:

❖ **Group A:** 30° insertion angle;
❖ **Group B:** 45° insertion angle;
❖ **Group C:** 90° insertion angle (perpendicular).

#### 2.2.2. Meshing and Contact Definitions

Meshing was performed in ANSYS Workbench 2023 R2 (ANSYS Inc., Canonsburg, PA, USA). Each model was meshed using tetrahedral elements, with local mesh refinement around the bone–screw interface. Each model contained approximately 200,000 elements and 230,000 nodes. Mesh convergence testing was performed to ensure solution accuracy.

A bonded contact was defined between the screw and the surrounding bone to simulate ideal primary stability, with no micromotion at the interface. The lower and lateral surfaces of the bone block were fully constrained in all directions.

#### 2.2.3. Loading Conditions

A horizontal orthodontic force of 2 N was applied at the head of the miniscrew in the labio-lingual direction, simulating clinical force conditions used during en-masse retraction. The same magnitude and direction of force were used in all groups for consistency.

### 2.3. Material Properties

All components in the model were assumed to be homogeneous, isotropic, and linearly elastic. The elastic modulus and Poisson’s ratio of the materials were derived from previously published studies [42,43] and are presented in Table 1.

### 2.4. Outcome Measures and Analysis

#### Quantitative Output

**Figure 2:**
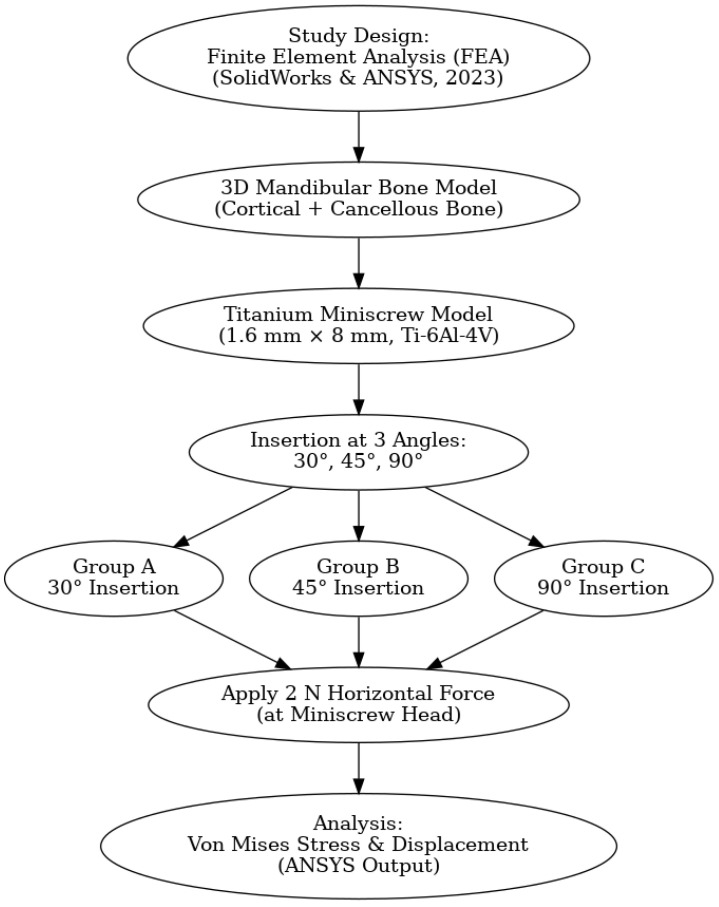
Flowchart of study.

From each simulation, maximum von Mises stress values (MPa) were extracted at the cortical and cancellous bone nodes. As these are deterministic peak outputs of the FEA, they are reported without standard deviation. For displacement, however, mean ± SD values were calculated across nodes of the miniscrew to reflect both the average deformation and its variability within the structure. In addition to quantitative outputs, color-coded stress distribution maps were generated to visually and qualitatively assess the patterns and localization of stress concentration. All simulations and post-processing analyses were conducted using the dedicated tools within ANSYS Mechanical (Figure 2).

### 2.5. Statistical Analysis

The finite element analysis generated quantitative data regarding von Mises stress (MPa) and displacement (mm) values for each insertion angle group (30°, 45°, and 90°). Descriptive statistics, including mean, standard deviation (SD), and maximum stress values, were calculated for each group using IBM SPSS Statistics version 26.0 (IBM Corp., Armonk, NY, USA). The normality of data distribution was assessed using the Shapiro–Wilk test. For normally distributed data, one-way analysis of variance (ANOVA) was performed to compare the stress and displacement values among the three insertion angles. If significant differences were detected, Tukey’s post hoc test was used for pairwise comparisons. For non-normally distributed data, the Kruskal–Wallis test was employed. A *p*-value of <0.05 was considered statistically significant for all tests.

## 3. Results

### 3.1. Von Mises Stress Distribution

The von Mises stress values varied significantly depending on the insertion angle of the miniscrew. The highest stress concentration was observed in the 90° group, with a maximum von Mises stress of 58.2 MPa, predominantly located at the cortical bone entry point. In contrast, the 45° insertion angle showed moderate stress levels (42.7 MPa), while the 30° group exhibited the lowest stress concentration (36.4 MPa) in both cortical and cancellous bone regions (Table 2).

In all three groups, stress was primarily localized in the cortical bone layer, particularly around the cervical threads of the miniscrew, while the apical portion within cancellous bone experienced relatively lower stress levels. The 90° insertion model showed a more concentrated and sharper stress peak at the cortical entrance, whereas the 30° and 45° angles resulted in a more diffuse stress distribution along the screw–bone interface.

**Figure 3:**
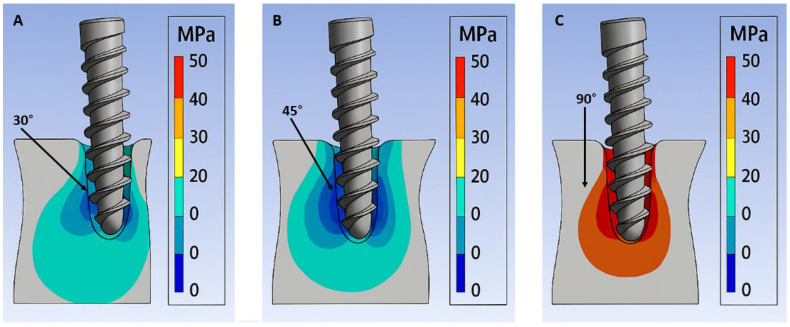
Color-coded von Mises stress distribution in alveolar bone according to miniscrew insertion angle. (**A**) 30° insertion shows moderately distributed stress along cortical bone with minimal cancellous stress. (**B**) 45° insertion produces moderate cortical concentration with broader stress dispersion. (**C**) 90° insertion results in localized peak stress at the cortical entry point around the first threads.

Color-coded stress distribution maps further supported these findings. The red zones, indicating peak stress, were most prominent at the cortical surface in the 90° group, whereas more gradual stress transitions were observed in the oblique angle models. These patterns are illustrated in Figure 3.

### 3.2. Displacement Analysis of the Miniscrew

**Figure 4:**
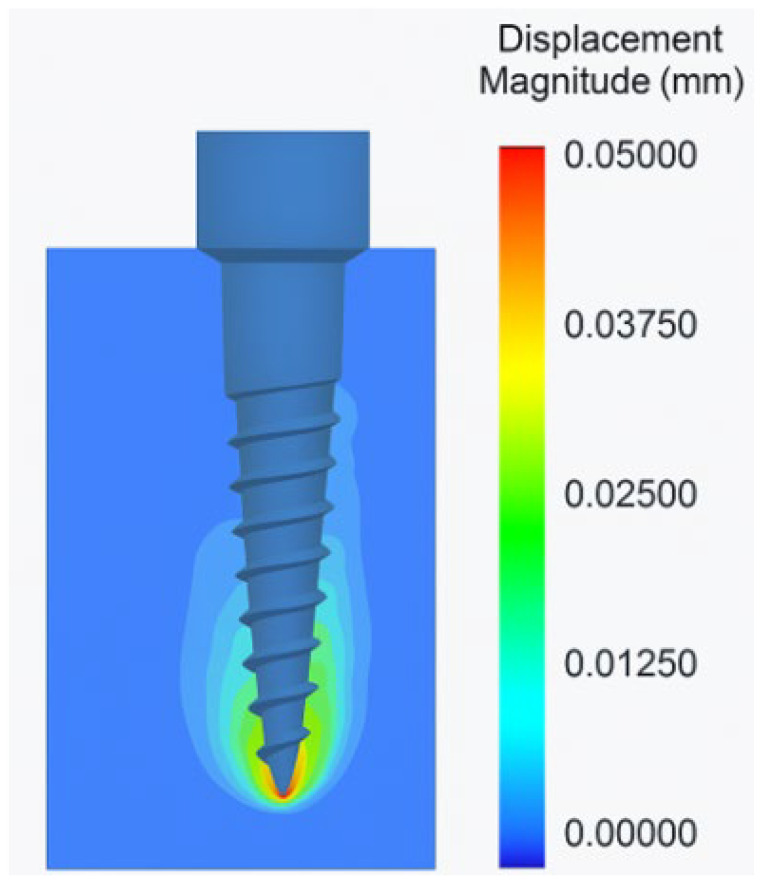
Displacement analysis of the orthodontic miniscrew under 2 N horizontal force applied at 90° insertion angle.

The analysis of miniscrew displacement under a horizontal orthodontic load of 2 N revealed significant variation based on insertion angle. The lowest total displacement was observed in the 90° group, with a mean value of 0.023 mm, indicating high mechanical resistance to deformation under loading conditions. In contrast, the 45° insertion angle resulted in a moderate displacement of 0.035 mm, while the 30° group demonstrated the highest displacement, with a mean value of 0.052 mm. As the insertion angle decreased from vertical (90°) to oblique (30°), the miniscrews exhibited increasing deflection, especially in the apical and cervical regions. This pattern suggests that oblique insertion may reduce primary mechanical stability, leading to greater susceptibility to micromotion under functional loading. The trend clearly indicated that more vertical insertion angles provided greater resistance to deformation, thereby enhancing mechanical stability (Table 3, Figure 4).

### 3.3. Comparative Evaluation of Insertion Angles

When the insertion angles were compared, both von Mises stress values and displacement magnitudes showed statistically significant differences among the three groups (*p* < 0.05). As shown in Table 4, the 90° insertion angle resulted in the highest von Mises stress values (58.2 MPa) but the lowest displacement (0.023 mm), indicating high mechanical rigidity but increased stress concentration at the cortical bone interface.

Conversely, the 30° angle generated the lowest stress levels (36.4 MPa), indicating a more favorable stress distribution within the surrounding bone; however, it was associated with the highest displacement value (0.052 mm), suggesting reduced resistance to deformation under functional loading. The 45° angle offered a biomechanical balance, with moderate stress (42.7 MPa) and displacement (0.035 mm), and could therefore represent a clinically optimal compromise between stress dissipation and mechanical stability (Table 4).

### 3.4. Visual Interpretation of Stress Patterns

The finite element models displayed variations in stress distribution depending on the insertion angle of the miniscrew. In the 30° and 45° insertion groups, stress distribution appeared more uniform, with smoother color gradients extending from the screw surface into the surrounding cortical bone. Color transitions were gradual, and stress levels were visibly distributed over a wider area. In the 90° insertion model, stress was concentrated at the cortical bone entry point, particularly around the first few threads of the screw. The intensity of the red region was higher compared to the other groups, indicating localized peak stress areas. Across all models, the cortical bone exhibited higher stress intensity, whereas the cancellous bone showed lower stress levels, represented by blue to green color ranges. The stress fields in oblique insertion angles (30° and 45°) extended further into the surrounding bone, while the vertical angle (90°) showed more localized stress accumulation. Displacement contours demonstrated a gradient distribution along the screw axis in all groups.

## 4. Discussion

This study utilized three-dimensional FEA to evaluate how varying insertion angles of orthodontic miniscrews—specifically 30°, 45°, and 90°—influence stress distribution in surrounding alveolar bone and the mechanical stability of the miniscrew–bone complex under orthodontic loading. Our findings offer both biomechanical and clinical insights and align with, or diverge from, several previously published studies. This discussion aims to contextualize the current results within the existing literature and provide a nuanced interpretation of the data. These findings are consistent with recent meta-analyses highlighting the importance of insertion angle in clinical outcomes [6,7]. Moreover, other FEA-based investigations in the orthodontic and orthopedic fields continue to validate angled placement as a means of optimizing stress distribution [10,13,17,35]. By expanding the comparative framework, our study adds quantitative evidence to this ongoing debate.

The results demonstrated that the 90° insertion angle generated the highest von Mises stress (58.2 MPa), primarily concentrated at the cortical bone entry point. In contrast, 45° and 30° angles resulted in lower stress levels (42.7 MPa and 36.4 MPa, respectively) with more diffused stress patterns across the screw–bone interface. Wilmes et al. reported that perpendicular miniscrew insertion increases localized stress due to a reduced contact area between screw threads and cortical bone [20]. Popa et al. and Paul et al. found that oblique insertion angles produce a more favorable stress distribution by increasing the surface area in contact with cortical bone, thus facilitating more even stress dissipation. Moreover, the concentration of stress in the 90° group was sharply localized around the first few threads, posing a potential risk for cortical microfractures or resorption over time [18,35]. Our results are in line with earlier investigations over a decade ago, which already highlighted the biomechanical trade-off between stress concentration and displacement at different insertion angles [20,35,41]. These pioneering studies laid the groundwork for current simulation-based analyses, and our study expands this framework by providing a direct quantitative comparison across three clinically relevant angulations. This reflects the stiff nature of Ti-6Al-4V alloy, which, while providing rigidity, tends to transfer concentrated stresses to the cortical bone, potentially compromising long-term stability. Li et al. highlighted the role of peak stress concentration in long-term miniscrew failure, especially in the cortical region [30].

Our study showed that while the 90° group exhibited the highest stress, it also demonstrated the lowest displacement (0.023 mm), suggesting superior primary mechanical resistance. In contrast, the 30° group had the highest displacement (0.052 mm), implying reduced rigidity under functional loads despite lower stress levels. These findings align with Romanec et al. reported that vertical insertion angles produce greater insertion torque and initial stability [15]. However, this comes at the cost of higher cortical stress, which may not be ideal in compromised bone conditions. Sarika et al. also observed that obliquely inserted miniscrews undergo more displacement but produce more physiologic stress patterns, suggesting a biomechanical trade-off between rigidity and load distribution [13]. In practical terms, greater displacement in the 30° group may increase the risk of micromotion, which has been implicated in early failure due to impaired osseointegration or bone remodeling, as supported by Nenen et al. [31].

The 45° insertion angle demonstrated intermediate stress (42.7 MPa) and displacement (0.035 mm) values, representing a potential biomechanical compromise. This angle provided sufficient resistance to deformation while maintaining a favorable stress distribution, indicating its suitability as a clinically optimal insertion angle. This suggests that even without altering the material itself, modifying the insertion trajectory of Ti-6Al-4V miniscrews can enhance stress dissipation at the bone–implant interface. Sahoo et al. and Lee et al. also found that 45° insertions optimize the balance between primary stability and physiological load transmission [10,17]. Such findings reinforce the idea that while 90° angles offer higher immediate stability, they may predispose the surrounding bone to stress-related complications. Conversely, shallow angles such as 30° reduce stress concentration but compromise rigidity, making 45° an ideal intermediate.

Based on our findings, clinicians should consider using oblique insertion angles (30–45°) to minimize stress concentration in the cortical bone, thereby reducing the risk of microfractures and implant loosening. However, when primary stability is of paramount importance—such as in immediate loading or poor bone quality cases—a carefully executed 90° insertion may be justified, provided that anatomical and biomechanical limitations are respected. Ultimately, the choice of insertion angle should be individualized, accounting for cortical bone thickness, bone density, anatomical constraints, and the direction of orthodontic forces. The 45° angle appears to offer the most balanced biomechanical profile and should be considered the first-line approach in most clinical scenarios.

While several FEA and experimental studies have explored the biomechanical behavior of orthodontic miniscrews, few have systematically compared insertion angles with a combined focus on both stress distribution and displacement under identical orthodontic loading. The present study advances existing knowledge by integrating these two outcome domains and evaluating them comparatively across three clinically relevant angles (30°, 45°, and 90°). Previous studies such as Wilmes et al. and Popa et al. indicated that oblique insertion angles (30–60°) are associated with lower stress concentrations due to increased cortical surface contact [18,20]. Our findings support this but further quantify the stress gradient differences across angles, demonstrating that 90° insertion leads to a more sharply localized peak stress (58.2 MPa) while 30° results in broader dissipation (36.4 MPa). This level of granularity in stress distribution has not been simultaneously reported for these three insertion angles in earlier studies. We not only confirmed the presence of lower stress in oblique insertions but also provided a side-by-side comparison using color-coded stress maps and von Mises values, clarifying how insertion angle modulates cortical stress peaks and dispersion patterns. This comparative visual and numerical assessment is lacking in many earlier FEA reports.

Unlike some earlier works that focused predominantly on stress patterns, our study highlights a biomechanical trade-off: while 90° insertion offers greater mechanical rigidity (minimal displacement: 0.023 mm), it also poses a higher risk of stress-related bone damage. In contrast, 30° angles provide more favorable stress profiles but with increased displacement (0.052 mm), potentially compromising primary stability. This distinction between stress and displacement outcomes has not been explicitly emphasized in previous literature. For example, Sarika et al. and Romanec et al. reported isolated findings related to stress or insertion torque but did not explore the concurrent mechanical behaviors across multiple angles [13,15]. Our study is among the few to concurrently analyze stress distribution and miniscrew displacement under uniform loading conditions, providing clinically relevant biomechanical insight into optimal angulation strategies. Future studies should evaluate a broader range of insertion angles (e.g., 15° or 60°) to determine whether the biomechanical trends observed here extend to more extreme trajectories. Additional parameters such as screw diameter, thread design, and surface treatment could also be tested to assess their interaction with insertion angle. Incorporating different biomaterials or coated titanium surfaces may provide insights into how material properties influence stress distribution. Moreover, applying multi-directional or cyclic loading conditions would more closely simulate clinical orthodontic forces. Experimental and in vivo validation remains crucial to translate FEA predictions into clinical protocols.

Some prior studies have focused on extreme angles—either perpendicular (90°) or shallow (30°)—with limited attention to 45° insertion. Our findings indicate that a 45° insertion offers a biomechanical equilibrium, combining moderate stress levels (42.7 MPa) with acceptable displacement (0.035 mm). While Sahoo et al. suggested similar outcomes, our study quantitatively supports this claim with finite element validation [17]. We position the 45° insertion as a clinically optimal compromise between stress concentration and stability, a conclusion that is rarely drawn explicitly in the literature.

Many previous studies have used retrospective clinical data or in vitro models, which, while valuable, are limited by patient-specific variables and inconsistent force application [21,22]. In contrast, our study utilizes standardized geometry, loading, and boundary conditions within a controlled FEA framework, offering reproducible and isolated comparisons of insertion angles without confounding factors. The current study offers purely mechanical and controlled comparisons across angles under identical conditions, allowing for a clearer understanding of how insertion angle independently influences biomechanics.

### Limitations of the Study

This study has some limitations. The finite element models were based on idealized bone geometry and assumed isotropic, homogeneous, and linearly elastic properties, which do not fully reflect the complex, anisotropic nature of human alveolar bone. Important anatomical structures such as the periodontal ligament, adjacent teeth, and soft tissues were excluded, potentially leading to an overestimation of localized stress or deformation. Only static, unidirectional forces were applied, whereas clinical forces are dynamic and multi-directional. A perfectly bonded bone–implant interface was assumed, neglecting micromotion, incomplete osseointegration, and bone remodeling. Another limitation is that bone tissue was modeled as linearly elastic, isotropic, and homogeneous. In reality, craniofacial bone exhibits anisotropic and heterogeneous behavior, particularly in regions such as buttresses, sutures, and the symphysis. This simplification was chosen for computational feasibility and comparability with previous studies, but it may underestimate localized stress distributions. Future studies incorporating anisotropic and region-specific bone properties will provide more accurate biomechanical predictions. Another key limitation is the lack of experimental or clinical validation; the results should be confirmed through in vitro, cadaveric, or in vivo studies. Finally, only three insertion angles (30°, 45°, and 90°) were examined, which may limit generalizability. Future investigations should also evaluate whether alternative biomaterials or coated titanium surfaces demonstrate similar biomechanical trends under varying insertion angles. These factors highlight the need for further validation to enhance the clinical applicability of the findings.

## 5. Conclusions

In conclusion, this study demonstrated that miniscrew insertion angle significantly influences stress distribution and stability at the titanium–bone interface. While 90° insertions offer superior rigidity with minimal displacement, they also produce concentrated stress peaks in the cortical bone, potentially jeopardizing long-term stability. Oblique angles, particularly 45°, distribute stresses more evenly while maintaining sufficient primary stability, representing a biomechanical compromise, as it balanced moderate stress levels (42.7 MPa) with acceptable displacement (0.035 mm), unlike the 90° angle (58.2 MPa, 0.023 mm displacement) that concentrated stress in cortical bone or the 30° angle (36.4 MPa, 0.052 mm displacement) that exhibited higher micromotion risk. From a biomaterials perspective, these results emphasize that the performance of Ti-6Al-4V orthodontic miniscrews is not solely dictated by alloy properties but also by the biomechanical conditions under which they are deployed. Careful optimization of insertion angle can therefore enhance the functional integration of miniscrews without requiring changes in material composition. These insights may guide both clinicians in surgical protocols and biomaterials researchers in developing next-generation anchorage devices that balance strength, stress dissipation, and biological compatibility.

## Figures and Tables

**Table 1:** Mechanical properties used in the finite element analysis.

Material	Young’s Modulus (GPa)	Poisson’s Ratio
Cortical bone	13.7	0.30
Cancellous bone	1.37	0.30
Titanium alloy	110	0.34

**Table 2:** Maximum von Mises stress values (MPa) observed in cortical and cancellous bone for each insertion angle.

Insertion Angle	Cortical Bone Stress (MPa)	Cancellous Bone Stress (MPa)	Total Max Stress (MPa)
30°	32.1	4.3	36.4
45°	37.5	5.2	42.7
90°	50.6	7.6	58.2

**Table 3:** Total displacement values (mm) of the miniscrew under 2 N orthodontic force for each insertion angle.

Insertion Angle	Mean Displacement (mm)	Standard Deviation (mm)
30°	0.052	0.004
45°	0.035	0.003
90°	0.023	0.002

**Table 4:** Comparison of von Mises stress and displacement values across different insertion angles.

Insertion Angle	Max Von Mises Stress (MPa)	Mean Displacement (mm)	*p*-Value
30°	36.4	0.052	<0.05
45°	42.7	0.035	<0.05
90°	58.2	0.023	<0.05

## Data Availability

The data supporting the findings of this study are available from the corresponding author upon reasonable request.
